# Atrial Fibrillation Associated with Heart Failure: Perspectives from Recent Large Clinical Studies and Translational Research

**DOI:** 10.3390/jcm12155066

**Published:** 2023-08-01

**Authors:** Sid Ahmed Bentounes, Arnaud Bisson, Laurent Fauchier

**Affiliations:** 1 Service de Cardiologie, Centre Hospitalier Universitaire Trousseau, Faculté de Médecine, Université François Rabelais, 37044 Tours, Francelfau@med.univ-tours.fr (L.F.); 2 Service de Cardiologie, Centre Hospitalier Universitaire Orléans, 45100 Orléans, France

Atrial fibrillation (AF) is a major public health issue. In 2010, there were 20.9 million men and 12.6 million women worldwide who had AF. The estimated incidence rates were, in 100,000 person-years, at 77.5 (95% CI, 65.2–95.4) for men and 59.5 (95% CI, 49.9–74.9) for women, and its prevalence about 600 per 100,000 [1,2]. Furthermore, it is estimated that 64.3 million people worldwide have heart failure (HF) [3]. In Western countries, its prevalence is estimated to be between 1000 and 2000 per 100,000 [4] and its incidence varies from 100 to 900 cases per 100,000 person-years [5]. HF is found in 34,000 per 100,000 individuals with AF, and AF is found in 42,000 per 100,000 patients with HF [6]. Due to the aging of the population, the incidence will increase significantly in the coming years. Consequently, the cost of AF is an increasing burden for most healthcare systems, accounting for one-third of hospital admissions for cardiac rhythm disorders and hospital admissions for AF [2]. HF is a major cause of death in patients with AF. This article presents an overview regarding some recent findings and concepts about the association of AF and HF, reported in several large clinical analyses, meta-analyses, or translational research studies published in recent months. Recent guidelines on the management of AF show the need for an integrated approach to arrhythmia treatment using the ABC ‘Atrial fibrillation Better Care’ pathway, based on three items: ‘A’, anticoagulation/avoid stroke; ‘B’, better symptom control; ‘C’, optimization of cardiovascular risk factors and comorbidities [2]. This is also a convenient way to organize the presentation of these recent findings.

**‘A’, anticoagulation/avoid stroke**

AF is usually considered to be the cause of about one-quarter of all strokes. In patients with AF, the risk is particularly high in the elderly. Patients with AF-related strokes are more recurrent and severe [2]. This results in more expensive care and significantly greater long-term disability and mortality than those whose strokes are not related to AF. In the case of concomitant AF and HF, the risk of stroke is increased and the CHA_2_DS_2_-VASc score is concomitantly increased. The large registry reported by Krittayaphong et al. indicated that the yearly incidence of HF in AF patients in Thailand was around 3% [7]. The factors associated with HF incidence were older age (>65 yo), female sex, previous HF, coronary artery disease, previous implantation of a cardiac electronic device, diabetes, smoking, hypertension, renal replacement therapy, and left ventricular (LV) ejection fraction (EF) < 50% [7]. Enlargement of the left atrial appendage is observed in two-thirds of AF patients with a dilated left atrium. LV diastolic dysfunction typically leads to an increase in LV filling pressure, which in turn causes left atrial dilation. This dilation, along with elevated pressure, leads to blood stasis, promoting the formation of spontaneous echo contrasts that predispose to thrombosis and stroke. The CHA_2_DS_2_-VASc score for risk stratification of thromboembolic events is the worldwide leading scoring system for patients with AF [2]. Criterion C of this system represents 1 point for congestive heart failure (CHF) defined as clinical HF or a LVEF lower than 40%. Left ventricular function gradually worsens when the CHA_2_DS_2_-VASc score increases in AF patients. Echocardiographic markers of altered diastolic function are also known for their association with the incidence of stroke. Left ventricular (LV) filling pressure is increased and diastolic properties are worsened with the elevation of CHA_2_DS_2_-VASc score in patients with AF [8]. In an analysis by Jang et al., CHA_2_DS_2_-VASc score categories showed a significant increase in the risk of thromboembolic events with increasing predicting value when HF was defined as E/E′ ≥ 11, but not with the original definition of heart failure for the C criterion of the score [8]. The inclusion of refined criteria for HF in the CHA_2_DS_2_-VASc score could thus be optimized to better stratify the thromboembolic risk in these patients. Oral anticoagulation is indicated in patients with AF and HF since the CHA_2_DS_2_-VASc score is at least 1, and NOACs are now a main treatment for stroke prevention considering their efficacy and convenient use, unless they are contraindicated [2]. The presence of HF may affect the treatment of AF in many aspects, and particularly, anticoagulation may not be optimally managed in these patients with a higher thromboembolic risk as reported in the Polish analysis by Gawalko et al. [9]. Hypertension further contributes to the development of complications including ischemic or haemorrhagic stroke in AF patients. Those with uncontrolled high blood pressure should be classified as “high-risk” and rigorous blood pressure management, along with OAC, should help lower the risk of ischemic stroke [2]. This overall suggests room for improvement in stroke prevention for patients with AF and HF.

**‘B’, better symptom control**

The management of simultaneous AF and HF may be complex and may vary according to clinical presentation [2,6]. Symptoms may include palpitations directly related to AF and functional limitations including dyspnea or fatigue related to both AF and HF. AF has an impact on atrial function. It also causes intracellular oxidative stress, leading to calcium overload and the onset of an inflammatory cascade. This results in the perpetuation of the arrhythmia and remodeling of the left atrium, most often resulting in fibrosis [10]. The combination of AF and HF and the order in which these two conditions occur may affect prognosis, which seems worse when HF precedes AF [6]. The review by Derndofer et al. emphasized that AF and HF have the potential to interfere with each other and proper selection and timing are crucial when implementing different treatment strategies in patients with AF and HF [11]. Early rhythm control (whether with antiarrhythmic drug or with catheter ablation) should be taken into consideration, as there is evidence that it is associated with better cardiovascular outcomes [11,12]. In the event of haemodynamic instability or worsening HF, electrical cardioversion should be performed as an emergency measure; otherwise, pharmacological cardioversion with intravenous amiodarone is preferred [2]. AF and HF mutually worsen prognosis by increasing the probability of complications when they occur together [2,10]. Both may be seen as the cause or consequence of the other and treating one condition may treat the other and may improve the general prognosis. Catheter ablation of AF in patients with HF increases the likelihood of restoring sinus rhythm, thus reducing palpitations and improving LV function, functional capacity, and symptoms of HF [2,11]. AF ablation in HF patients is crucial for improving quality of life, reducing HF hospitalizations, and reducing deaths, as mentioned above, provided that LVEF >= 25% [11]. The general approach to managing AF may markedly differ between HF and non-HF patients. Anticoagulation is the cornerstone of treatment when the risk of stroke is not truly low, which is the case when HF is present. However, maintaining sinus rhythm and controlling heart rate usually require a patient-specific strategy based on the duration of AF, left atrial dimensions, and anticipated adverse effects of anti-arrhythmic drugs (AADs) [2]. This is partly related to common risk factors and pathophysiological phenomenons including structural cardiac remodeling, activation of neurohormonal mechanisms, and impairment of LV function related to tachycardia or ventricular irregularity [9]. Regarding rhythm control management in AF associated with HF, it has been shown that compared to non-invasive treatment, catheter ablation is often associated with more satisfactory results. The comprehensive meta-analysis by Magnocavallo et reported that AF ablation is more effective than conventional drug therapy when evaluating AF recurrence, improvement of LVEF, peak oxygen consumption (VO2), and ultimately improving survival [13]. The pooled analysis by Androulakis et al. also evaluated the possible benefits of rhythm control in AF patients in the specific setting of HF with preserved EF using AF catheter ablation. The increase in afterload caused by aldosterone results in additional myocardial fibrosis, and its levels have been shown to decrease after successful cardioversion of AF and this benefit might also be present when AF ablation is efficient [13]. New screening methods for AF in HF patients would also be relevant in the future due to the higher risk of expected complications when AF is diagnosed. In particular, artificial intelligence is an efficient method able to learn from ECGs and perform a self-diagnosis of AF. This approach seems capable of predicting the risk of subsequent AF from ECGs in sinus rhythm [14]. This would provide interesting tools to identify individuals at risk of developing AF for tailored management and possibly a better primary prevention of AF and its symptoms.

**‘C’, cardiovascular risk factors and comorbidity optimization**

AF and HF share common comorbidities and are linked for many reasons, both pathophysiological and biological. AF and HF share risk factors such as older age, hypertension, overweight and obesity, diabetes, and/or coronary artery disease [7]. In this context, regular visits to the general practitioner or cardiologist should be associated with a better prognosis in patients with AF and other cardiovascular problems, including hypertension, obesity, diabetes, or sleep apnoea syndrome, for example. According to the 2020 guidelines, therefore, careful monitoring can have significant benefits for these individuals [2]. This may include dietary/lifestyle management as well as education to physicians and patients, possibly using systems approaches (e.g., electronic medical record-based interventions or messaging) to improve control of multiple risk factors and management of comorbidities [15]. The causes of death in patients with AF are HF in 14.5%, malignancy in 23.1%, sepsis in 17.3%, and stroke in 6.5% [16]. HF and AF are two interrelated conditions. For this reason, it is recommended that patients with HF should be closely followed using regular ECGs to diagnose AF as early as possible and with an integrated management of comorbidities when AF is diagnosed [2].

There are many pathophysiological similarities between AF and HF (some of them also resultfrom the comorbidities mentioned above), with structural remodeling and fibrosis observed in both the atrial and ventricular myocardium, leading to changes in electrical conduction and diastolic dysfunction. HF with preserved ejection fraction (EF) is the presence of signs of pulmonary congestion while LVEF is above 50% and is now the most common form of HF when compared to HF with reduced EF, increasing by 10% every decade [17]. In an analysis by Ma et al., atrial remodeling and atrial fibrosis were the main morphological bases of AF [18] and this may be particularly relevant in patients with HF and preserved EF. Transforming growth factor β1 (TGF-β1) and NADPH oxidase (Nox4) are necessary for fibrogenesis [18]. This process may promote the accumulation of collagenous deposits, significant proliferation of atrial fibroblasts, and may aggravate atrial structural remodeling (ASR) [19] possibly resulting in the development of AF. Therefore, inhibition of TGF-β1 and Nox4 holds promise to reduce atrial fibrosis and AF. Intermedin1-53 (IMD1-53) may reduce atrial fibrosis as well as the inducibility and duration of AF in rats with HF. Intermedin (IMD) is a prepropeptide of 148 amino acids, encoded by the human IMD gene, comprising an N-terminal signal peptide for secretion. IMD1-53 is produced by proteolysis of the IMD and is expressed in the whole body, particularly in the cardiovascular system. It plays a role in vascular homeostasis and may have protective functions including cardiac protection, blood pressure regulation, increased angiogenesis, and anti-apoptotic effects. It also has an anti-fibrosis effect on pulmonary and renal fibrosis [20]. Overexpression of TGF-β1 in animal models and individuals with diagnosed AF intensifies the development of arrhythmia related to cardiac structural disorganization and dysfunction associated with fibrosis [21]. The recent analysis by Ma et al. interestingly reported that the suppression of TGF-β1 can effectively prevent atrial fibrosis and decrease susceptibility to AF [18]. These may be promising perspectives for primary prevention of AF and AF-related HF associated with cardiovascular comorbidities in the future.

**Conclusions**

HF and AF are two strongly related conditions that mutually worsen their prognosis when they occur together. To improve the management of these patients, several avenues are being explored. Treatment through catheter ablation appears to be one of the most promising approaches at the present time due to its relatively long-term effect compared to the frequent lack of adherence and persistence in drug treatment among patients. For patients with HF, the use of artificial intelligence in medicine could be relevant in the future. By developing an algorithm based on clinical characteristics and different ECGs, a computer might be able to perform a self-diagnosis of AF. This will allow clinicians to closely monitor HF patients and promptly manage AF if it occurs. It will also assist clinicians in predicting the occurrence of AF in HF patients and, conversely, in preventing complications and improving prognosis using a holistic approach of stroke prevention, management of symptoms including palpitations and functional capacity, and treatment of associated comorbidities.
